# Radiogenomic profiling to determine BRCA alteration status—a systematic review and meta-analysis

**DOI:** 10.1093/bjr/tqaf139

**Published:** 2025-06-25

**Authors:** David McAuliffe, Matthew G Davey, Michael J Kerin

**Affiliations:** School of Medicine, University of Galway, Galway H91 TK33, Ireland; School of Medicine, University of Galway, Galway H91 TK33, Ireland; Department of Surgery, Galway University Hospitals, Galway H91 YR71, Ireland; School of Medicine, University of Galway, Galway H91 TK33, Ireland; Department of Surgery, Galway University Hospitals, Galway H91 YR71, Ireland

**Keywords:** BRCA, radiomics, radiogenomics, breast cancer, ovarian cancer

## Abstract

**Objectives:**

Approximately 10% of breast and 20% of ovarian cancers are hereditary in nature. The most commonly implicated genes are the BRCA genes, and the current gold standard for testing is by direct DNA sequencing. This process is expensive, time-consuming, and has a turnaround time of several weeks. Radiogenomics involves extracting quantitative data from medical imaging and using mathematical models to predict the underlying genetic makeup of tissues.

**Aim:**

To perform a systematic review and meta-analysis evaluating the accuracy of radiogenomics in determining BRCA alteration status.

**Methods:**

A systematic review was performed in accordance with PRISMA guidelines. Diagnostic test accuracy analyses (i.e. pooled sensitivity and specificity) were performed. Statistical analyses were performed using RevMan V5.4.

**Results:**

Thirteen studies compromising 2835 patients were included. Of these, 857 were BRCA alteration carriers. The mean age of patients was 46 years. Radiogenomic methods correctly identified BRCA alteration with a strong diagnostic test accuracy (pooled sensitivity: 0.82, 95% confidence interval [CI]: 0.79-0.84, pooled specificity: 0.81, 95% CI: 0.78-0.83).

**Conclusions:**

Radiogenomics may be an accurate method to predict BRCA alterations. However, these findings should be validated in larger, prospective studies to determine their utility in clinical practice. Until further refinement of these methods, DNA sequencing should remain the gold standard.

**Advances in knowledge:**

To the best of our knowledge, this is the first systematic review and meta-analysis that has been carried out on this topic. We believe that our results demonstrate the potential clinical utility radiogenomics could have in the BRCA alteration testing process.

## Introduction

Hereditary breast and ovarian cancer syndrome (HBOC) is a condition that increases the risk of developing cancer of the breast, ovary, and other solid organs.^1^ While there are a number of gene alterations implicated in the hereditary development of breast cancer, the BReast CAncer genes (BRCA) 1 and 2 are the most commonly implicated.^2^ Thus, formalized testing for BRCA alterations in this population is now commonplace in clinical practice. The gold standard analytical method is direct (Sanger) DNA sequencing which has been used for over 2 decades.^3^ This method is expensive and time-consuming, and with an ever-increasing demand for testing, clinical laboratories are currently at risk of becoming overwhelmed.^4^ Newer methods such as next-generation sequencing have been developed in recent years to improve the speed and efficiency of genetic testing.^4^

Detecting deleterious alterations of these genes is important in both the prognosis and management of those affected by breast or ovarian cancer and for their unaffected relatives. Overall, there is a cumulative risk of breast cancer to 80 years of age of 72% for women with BRCA1 alterations and 69% for BRCA2 alterations and for ovarian cancer, the cumulative risk to age 80 years is 44% for BRCA1 and 17% for BRCA2.^5^ A meta-analysis by Chen et al^6^ found that there was a higher association with triple negative breast cancer, higher grade and larger tumour burden in women who had the BRCA1 mutation compared to those with sporadic disease. Conversely, studies have shown that hereditary ovarian cancers due to the BRCA gene have a more favourable prognosis, with better response to platinum-based therapies, and they can be targets for poly(ADP-ribose) polymerase (PARP) inhibitor therapy.^7^^,^^8^ In women with confirmed BRCA gene alteration, risk-reducing surgery in the form of prophylactic mastectomy and salpingo-oopherectomy has been shown to be effective in reducing cancer.^1^

Personalized medicine is an emerging approach within oncology which involves the tailoring of therapeutic decision making in accordance with the genetic profile of each patient.^9^ A technological advancement which is changing the landscape of personalized medicine is radiomics. “Radiomics”, first proposed by Lambin et al,^10^ is a rapidly growing field of precision medicine that involves extracting large amounts of data from medical images, which is imperceptible to the human eye, and applying this data to advanced mathematical models to enhance diagnosis, prognosis and to aid in clinical decision making.^11^ Radiomic analysis is a complex multi-step process but the general workflow involves: image acquisition, image preparation and segmentation, feature extraction, feature selection, model validation and performance evaluation.^11^ Its most significant application has been within the field of oncology, where the more specific term “radiogenomics” is used when the imaging features relate to underlying gene expression.^12^ However, across the literature the terms “radiomics” and “radiogenomics” tend to be used interchangeably. Since the terms were originally coined there has been an exponential increase of their appearance in the literature, with over 1500 publications in 2020.^11^

Solid tumours are heterogeneous, both spatially and temporally, and conventional biopsy may miss clinically significant information. One of the potential advantages of radiogenomics is that medical imaging has the ability to capture the heterogeneity of the entire tumour, non-invasively.^10^ Thus, the application of radiogenomics to inform genetic alterations may further personalize therapeutic and surgical strategy in accordance with the respective needs of each patient.

In contemporary practice, patients are selected for BRCA testing based on prediction models which take into consideration age and family cancer history; however, information about family structure may be deficient and this could potentially preclude those with a genetic susceptibility from testing.^13^ By using radiomics to reliably extract imaging features which are consistent with BRCA1/2 alterations, it may help improve selection of patients for genetic testing, which could subsequently expedite accurate diagnoses of BRCA alterations within this population, years prior to those alterations giving rise to breast and ovarian cancer diagnoses.^14^ Accordingly, the aim of this study was to perform a systematic review and meta-analysis to evaluate the diagnostic ability of radiogenomic profiling to accurately predict alterations in the BRCA gene.

## Methods

### Study design

A systematic review was performed in accordance with the Preferred Reporting Items for Systematic Reviews and Meta-analyses (PRISMA) guidelines^15^ and the Cochrane Handbook for Systematic Reviews of Diagnostic Test Accuracy.^16^ This study was prospectively registered with the International Prospective Register of Systematic Reviews (PROSPERO—CRD42023476196). Ethical approval for this study was not required as the outcome data has been published previously.

### Population, intervention, comparison, and outcome

The population, intervention, comparison, and outcome (PICO) framework was used to formulate the research question.^17^

1. Population: Patients who have a confirmed alteration of the BRCA1 or 2 gene by genetic testing and who have undergone radiomic analysis of the breast or ovary.
2. Intervention: Detection of alterations in the BRCA gene by radiogenomic profiling.
3. Comparison: Detection of alterations in the BRCA gene through direct DNA sequencing or next-generation sequencing.
4. Outcomes: The evaluation of the accuracy, and thus clinical utility, of radiomics to detect alterations in the BRCA gene as evidenced by the diagnostic parameters of sensitivity and specificity.

### Search strategy

An electronic search of the databases PubMed Medline, EMBASE, and Scopus was initially performed on February 8, 2023 with a further updated search carried out on February 20, 2025. The searches were carried out by 2 independent reviewers (DMcA and MGD). The searches used the following terms: (BReast CAncer gene) OR (BRCA) AND (Radiomics) OR (Radiogenomics). Searches were conducted from inception to February 2025. Only studies published in the English language were included. Retrieved results were exported to EndNote20 prior to screening. Once duplicate studies were removed, the titles and abstracts of all remaining studies were screened according to inclusion criteria. A third reviewer (MJK) acted as an arbitrator where there was discrepancy in opinion between the 2 primary reviewers. All potentially eligible studies subsequently had their full texts reviewed while applying our inclusion and exclusion criteria. Included studies had their reference lists screened to ensure any additional relevant studies were captured.

### Inclusion and exclusion criteria

Inclusion and exclusion criteria along with a data collection proforma were agreed upon by both reviewers and the senior author prior to commencement of the study. Studies which met the following inclusion criteria were included in the review: (1) Studies whose primary goal was to evaluate the ability of radiogenomic characterization algorithms to predict BRCA gene alteration status, (2) studies involving the ovarian and breast region only, (3) studies which used any conventional imaging modality were considered (mammography, ultrasound [US], computed tomography [CT], magnetic resonance imaging [MRI], positron emission tomography [PET]), (4) studies detailing sensitivity, specificity or receiver operating characteristic (ROC) curve analyses assessing the diagnostic capability in predicting BRCA alterations, and (5) studies published in English. While only studies which provided results allowing for the analysis of sensitivity and specificity data were eligible for inclusion in the meta-analysis, we included all relevant literature pertaining to the application of radiomic profiling to decipher BRCA alterations in the systematic review component of this study.

The exclusion criteria included: (1) studies which used imaging characteristic without the use of radiogenomics (i.e. radiologist opinion, contrast uptake parameters, etc.) to determine BRCA alteration status, (2) subsequent studies involving the same patient population, (3) conference abstracts, and (4) case reports.

### Data extraction and quality assessment

The following data was extracted from the eligible studies into the data collection proforma: (1) first author name, (2) year of publication, (3) study design, (4) country, (5) level of evidence, (6) number of participants, (7) age demographics, (8) number of BRCA positive alteration participants, (9) imaging modality used, (10) region of interest, and (11) sensitivity, specificity, and area under the curve (AUC) scores from ROC analyses, where available. Sensitivity and specificity were directly extracted from tables and study text. Where studies tested multiple radiogenomic methods, only data relating to the best performing method was extracted. In studies where participants were separated into training and testing cohorts, results from the testing cohorts were used, to minimize potential bias which may arise from the training cohorts. Similarly, where studies used age-matched cohorts, results from these cohorts were extracted. An appraisal of the quality and risk of bias of the included radiogenomic studies was carried out independently by 2 reviewers (DMcA and MGD) and assessed using the radiomics quality score (RQS) as described by Lambin et al^18^ and the Quality Assessment of Diagnostic Accuracy Studies 2 (QUADAS-2).^19^ Description of these tools along with results included in this study can be found in the Supplementary Material. Any discrepancies between reviewers were resolved following discussion with final judgement by the senior author (MJK).

### Statistical analysis

Descriptive statistics were used to outline characteristics of the various included studies. Statistical analysis was performed in accordance with the Cochrane guidelines.^16^ Study specific estimates of sensitivity and specificity were calculated from the study data. Summary ROC analysis was used to convey the relationship between sensitivity and specificity of radiogenomic analysis in detecting BRCA gene alteration and to demonstrate the diagnostic test performance of radiogenomics in detecting BRCA gene alteration. Where sensitivity and specificity were not explicitly stated by the authors, estimated diagnostic test sensitivity and specificity were calculated from ROC analyses with the most accurate sensitivity prioritized. For all sensitivity and specificity analyses, 95% percent confidence intervals (CI) are reported. Statistical significance was determined to be a *P*-value <.05. No heterogeneity test was applied as sensitivity and specificity analysis did not lend the opportunity for such analysis. Statistical analysis was undertaken using Review Manager (RevMan), version 5.4 (Nordic Cochrane Centre, Copenhagen, Denmark).

## Results

### Literature search

**Figure 1.**
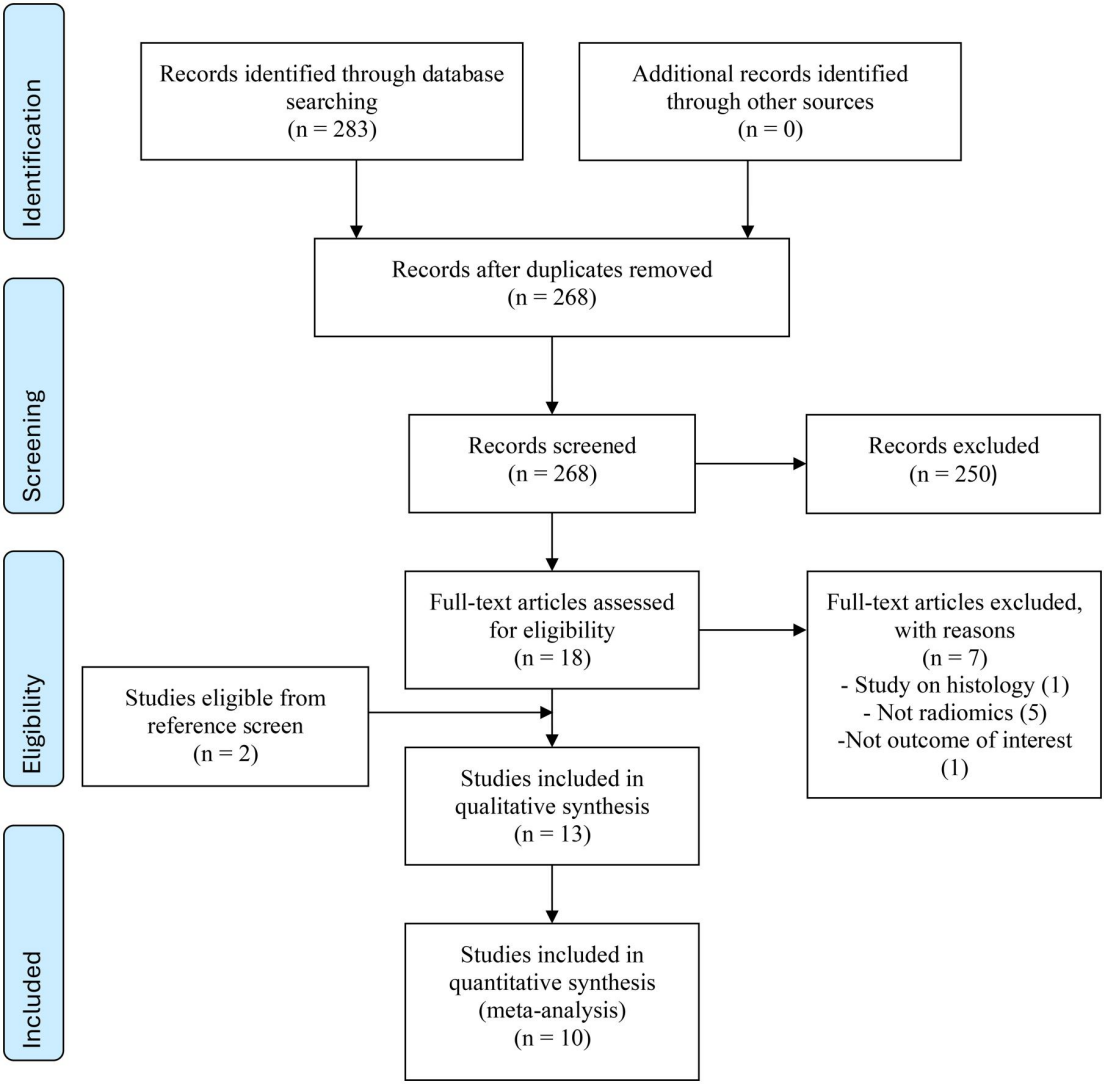
PRISMA flowchart outlining systematic literature search.

The initial electronic database search yielded a total of 283 studies of which 15 were duplicates, leaving 268 to be reviewed. After screening of titles and abstracts, 18 manuscripts were deemed appropriate for full text and reference screening. From this, 11 studies were deemed to meet the inclusion criteria and 2 further studies were identified in the reference screening, resulting in a total of 13 studies included in the systematic review.^14^^,^^20–31^ Thereafter, we noted that 10 of those provided data which was eligible for inclusion in the meta-analysis.^14^^,^^20–23^^,^^25^^,^^26^^,^^28^^,^^30^ The literature search did not produce any non-English language papers that the authors felt could have potentially relevant results. The PRISMA flow chart of study selection is depicted in Figure 1.

### Study characteristics and quality of studies

The 13 studies included had a total of 2835 patients, of which 1255 were used in the test and age-matched cohorts. There were 857 BRCA alteration carriers. The mean age was 46 years (range: 19-89 years) (10 studies). Seven of the studies looked at radiogenomic analysis of the breast, while 6 looked at ovarian tissue. There was a combination of both healthy tissue and tumour for both regions of interest. The majority of the studies were conducted in China (46%). All of the included studies were a retrospective cohort design. Quality of the included studies was satisfactory with the mean RQS being 17.6/36 (range 12-21). While the retrospective nature of the included studies added the potential for bias, using the QUADAS 2 tool it was deemed that overall the risk for bias was low. Tables 1 and 2 provide a summary of the study characteristics.

**Table 1.** Study demographics.

Author	Year	Design (LOE)	Country	Total no. of participants	No. in test/age matched group	Mean age in years (range)	No. BRCA+
Avesani et al^20^	2022	Retrospective (III)	Italy	218	66	56.4 (29-86)	73
Cao et al^21^	2023	Retrospective (III)	China	96	28	53 (42-62)	41
Deng et al^22^	2024	Retrospective (III)	China	497	149	43.2 (33-53)	120
Du and Zhao^23^	2022	Retrospective (III)	China	125	25	55.4 (NR)	89
Gierach et al^24^	2014	Retrospective (III)	USA	237	60	48.3 (23-79)	137
Guo et al^25^	2023	Retrospective (III)	China	449	138	42.5 (33-53)	92
Huo et al^26^	2002	Retrospective (III)	USA	172	90	44.3 (33-55)	30
Li et al^27^	2017	Retrospective (III)	USA	456	381	55.8 (21-89)	53
Li et al^28^	2021	Retrospective (III)	China	95	N/A	57 (36-79)	37
Meier et al^29^	2019	Retrospective (III)	USA	88	N/A	NR (32-82)	28
Mingzhu et al^30^	2021	Retrospective (III)	China	106	30	54.4 (35-77)	43
Nero et al^31^	2020	Retrospective (III)	Italy	255	64	45.4 (23-79)	98
Vasileiou et al^14^	2020	Retrospective (III)	Germany	41	N/A	37.5 (19-68)	16

Abbreviations: LOE = level of evidence; NA = not applicable; NR = not reported.

**Table 2.** Study image acquisition characteristics and RQS.

Author	Year	Imaging modality	Imaging protocols/views	ROI	Non-imaging variable	RQS
Avesani et al^20^	2022	CT	Abdomen/pelvis—PV	Ovarian tumour	Y	18
Cao et al^21^	2023	CT	Abdomen/pelvis—A, PV	Ovarian tumour	Y	19
Deng et al^22^	2024	US	High frequency linear array probe	Breast tumour	Y	19
Du and Zhao^23^	2022	MRI	DCE, DWI	Breast tumour	N	19
Gierach et al^24^	2014	MMG	CC	Retroareolar	N	15
Guo et al^25^	2023	US	High frequency linear array probe	Breast tumour	Y	19
Huo et al^26^	2002	MMG	CC	Retroareolar	N	I/C
Li et al^27^	2017	MMG	CC	Retroareolar	N	12
Li et al^28^	2021	CT	Abdomen/pelvis- A, PV, delayed phase	Ovarian tumour	N	19
Meier et al^29^	2019	CT	Abdomen/pelvis- PV	Ovarian tumour	N	13
Mingzhu et al^30^	2021	CT	Abdomen/pelvis -A, PV, delayed phase	Ovarian tumour	N	19
Nero et al^31^	2020	US	TV	Healthy ovary	N	19
Vasileiou et al^14^	2020	MRI	fl3d with contrast	Breast tumour	Y	21

Abbreviations: A = arterial phase; CC = craniocaudal; CT = computed tomography; DCE = dynamic contrast enhanced; DWI = diffusion weighted imaging; fl3d = 3D fat-suppressed fast low angle shot; MMG = mammography; MRI = magnetic resonance imaging; PV = portal venous phase; ROI = region of interest; RQS = radiomics quality score; TV = transvaginal.

### Radiological imaging modalities

The imaging modalities utilized included CT, MRI, mammography and US. Five studies^14^^,^^20–22^^,^^25^ included a non-imaging variable along with radiogenomic analysis in their results, while Li et al^28^ used routine CT features in conjunction with radiogenomic analysis.

### Diagnostic accuracy of radiogenomics by modality

Two studies utilized MRI and looked at radiogenomic analysis of breast tumours.^14^^,^^23^ These studies had sensitivities and specificities which ranged from 0.76 to 0.84 and 0.64 to 0.83, respectively.

Five of the studies employed CT and assessed ovarian tumours,^20^^,^^21^^,^^28–30^ with sensitivities and specificities ranging from 0.60 to 0.83 and 0.55 to 0.90, respectively.

Three studies employed mammography to evaluate breast tissue.^24^^,^^26^^,^^27^ These studies looked at the retroareolar area of healthy breasts.

Three studies used US based radiogenomics. Nero et al^31^ undertook radiogenomic analysis of healthy ovaries. They used a number of different radiogenomic strategies and models of US machine. Reported sensitivities and specificities for the various strategies and machines used ranged from 0.18 to 0.75 and 0.48 to 0.91, respectively. The other two US studies looked at breast tumour.^22^^,^^25^ A summary of the data for the sensitivity and specificity analysis for studies included in the meta-analysis is illustrated in Table 3.

**Table 3.** Data for sensitivity and specificity of studies included in meta-analysis.

Study	TP	FP	FN	TN	Sensitivity (95% CI)	Specificity (95% CI)
Avesani et al^20^	53	30	13	36	0.80 (0.69,0.89)	0.55 (0.42,0.67)
Cao et al^21^	80	10	16	86	0.83 (0.74,0.90)	0.90 (0.82,0.95)
Deng et al^22^	400	137	87	360	0.82 (0.78,0.85)	0.72 (0.68,0.76)
Du and Zhao^23^	21	9	4	16	0.84 (0.64,0.88)	0.64 (0.43,0.82)
Guo et al^25^	395	60	95	430	0.81 (0.77,0.84)	0.88 (0.85,0.91)
Huo et al^26^	80	10	10	80	0.89 (0.81,0.95)	0.89 (0.81,0.95)
Li et al^27^	305	76	88	293	0.78 (0.73,0.82)	0.79 (0.75,0.83)
Li et al^28^	74	24	17	67	0.81 (0.72,0.89)	0.74 (0.63,0.82)
Mingzhu et al^30^	18	3	12	27	0.60 (0.41,0.77)	0.90 (0.73,0.98)
Vasileiou et al^14^	31	7	10	34	0.76 (0.70,0.88)	0.83 (0.68,0.93)

Abbreviations: CI = confidence interval; FN = false negative; FP = false positive; TN = true negative; TP = true positive.

### Pooled diagnostic accuracy of radiogenomics

**Figure 2.**
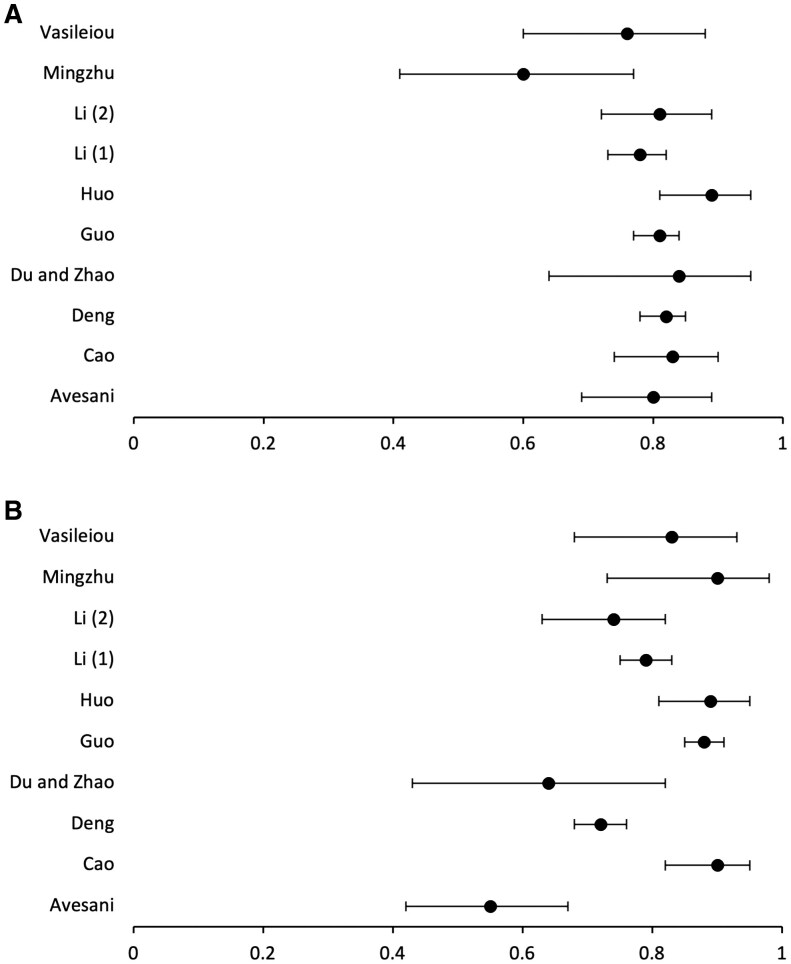
Individual studies sensitivity (A) and specificity (B) with 95% confidence intervals.

**Figure 3.**
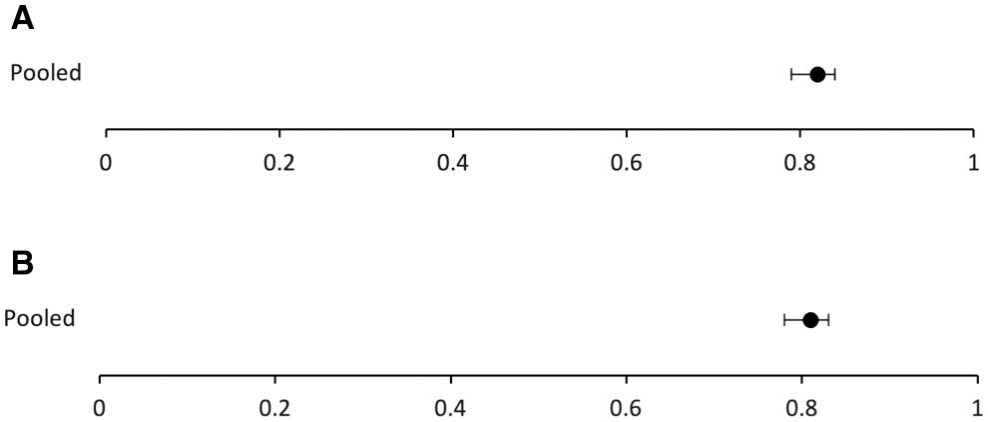
Pooled sensitivity (A) and specificity (B) for the meta-analysis with 95% confidence intervals.

The overall pooled sensitivity and specificity for studies included in the meta-analysis was 0.82 (95% CI 0.79-0.84) and 0.81 (95% CI 0.78-0.83), respectively. Figure 2 depicts the forest plots for sensitivity (Figure 2A) and specificity (Figure 2B) of the individual studies. Figure 3 depicts the forest plots for sensitivity (Figure 3A) and specificity (Figure 3B) of the pooled data.

## Discussion

This is the first systematic review which concerns the diagnostic ability of radiogenomics to predict alterations in the BRCA genes. Importantly, 12 of the included studies showed positive results in predicting BRCA alterations using radiogenomic analysis. The study conducted by Meier et al^29^ assessed associations between texture metrics and BRCA alteration status within ovarian tumours, but they were unable to discriminate between BRCA alteration and non-alteration carriers. They noted the small study population (*n* = 88) and BRCA subpopulation (*n* = 28) as possible contributors to these findings. At meta-analysis, radiogenomics correctly identified BRCA alteration status with a strong diagnostic test accuracy, having a pooled sensitivity and specificity of 0.82 and 0.81, respectively. Vasileiou et al,^14^ Avesani et al,^20^ Cao et al,^21^ Deng et al,^22^ and Guo et al^25^ combined non-imaging variables to improve their predictive performance, a technique that has been described elsewhere.^32^^,^^33^ The utilization of non-imaging variables is also captured in the RQS, resulting in a higher score, representing enhanced radiomic quality. This reinforces the fact that radiogenomics should be viewed as complementary to our current practice, and not as a replacement, in providing diagnostic, prognostic and predictive value.

In the broader field of radiomics, there are a number of published systematic reviews which have shown its clinical utility in a variety of settings. These include: predicting the response of breast cancer to neo-adjuvant chemotherapy with MRI,^34^ predicting distant metastases post-surgical resection in rectal carcinoma^35^ and in differentiating between molecular subtypes of breast cancer.^36^

Medical imaging is a fundamental part of modern medicine and given that radiogenomics has been shown to be both a cost-effective and non-invasive method of characterizing underlying tissue phenotype, it is feasible that it could have more widespread application in BRCA alteration testing.^37^ One of the most cumbersome aspects of radiomics is the manual segmentation step; however, with developments in artificial intelligence, this laborious task is having to be used less frequently and this will greatly increase the speed and improve the reproducibility of results.^38^

The National Cancer Control Programme (NCCP), whose role involves the prevention and treatment of cancer along with increasing survival rates and quality of life for those with cancer in the Republic of Ireland, recently published their Hereditary Cancer Model of Care.^39^ This publication recommends a dedicated national database pertaining to BRCA alteration carriers undergoing follow-up. This database, should it be developed and within the confines of General Data Protection Regulation (GDPR), could be used to help train future radiogenomic models and to develop a robust “radiomic signature” to improve their overall accuracy in predicting BRCA alteration status. This new model could then potentially be incorporated into The National Breast Cancer Screening programme (BreastCheck) in Ireland. This may subsequently facilitate earlier BRCA alteration status detection and these earlier detection rates could be translated into enhanced oncological and survival outcomes for many patients harbouring BRCA alterations, who may otherwise have delayed diagnoses. The implementation of radiomics in cancer screening programmes has previously shown promising results.^40^

There are a number of limitations to this study. Firstly, all of the included studies were single-centre and retrospective in design, which are known to be more prone to selection and confounding biases when compared to prospective studies.^41^ Secondly, the relatively small population size limits the power of the results. Finally, the included studies were conducted using different imaging modalities and across 2 distinct anatomical regions of interest, thus limiting interpretation somewhat. Furthermore, radiomics is still a heterogeneous area of study and involves a wide spectrum of different techniques to build models such as machine-learning and neural networks. In this study, we have combined all radiogenomic studies irrespective of model used; however, it is recognized that there can be variations in the reproducibility of data from these different techniques.^42^ Nevertheless this study provides a comprehensive review of studies using radiogenomics to aid the detection of BRCA alterations with strong diagnostic test accuracy, which broadens the horizon for further studies to refine this process, moving its application closer to use in clinical practice.

## Conclusion

Radiogenomics is a rapidly evolving field which has the potential to revolutionize personalized medicine. This review provides evidence for the use of radiogenomics in identifying BRCA alteration status. We recommend that our findings be validated in larger, prospective studies. Although currently direct DNA sequencing remains the gold standard for genetic testing, in the future radiogenomics may act as an adjunct to patient selection and, as technology advances, may become a validated diagnostic tool in its own right.

## Supplementary Material

tqaf139_Supplementary_Data

## Data Availability

Data will be made available upon reasonable request from the corresponding author.
