# Supplementary material for: Radiogenomic profiling to determine BRCA alteration status—a systematic review and meta-analysis
Source: Br J Radiol. 2025 Jun 25;98(1173):1383–9. doi: 10.1093/bjr/tqaf139 (PMC12377466; doi:10.1093/bjr/tqaf139)
Supplement: tqaf139_Supplementary_Data [file tqaf139_supplementary_data.docx]

**Supplementary material S1:** Description of the radiomics quality score (RQS) tool.

|  | **Criteria** | **Points** |
| --- | --- | --- |
| **1** | **Image protocol quality** - well-documented image protocols (for example, contrast, slice thickness, energy, etc.) and/or usage of public image protocols allowreproducibility/replicability | + 1 (if protocols are well-documented)  +1 (if public protocol is used) |
| **2** | **Multiple segmentations** - possible actions are: segmentation by different physicians/algorithms/software, perturbing segmentations by (random) noise, segmentation at different breathing cycles. Analyse feature robustness to segmentation variabilities | + 1 |
| **3** | **Phantom study on all scanners** - detect inter-scanner differences and vendor-dependent features. Analyse feature robustness to these sources of variability | + 1 |
| **4** | **Imaging at multiple time points** - collect images of individuals at additional time points. Analyse feature robustness to temporal variabilities (for example, organ movement, organ expansion/ shrinkage) | + 1 |
| **5** | **Feature reduction** or adjustment for multiple testing - decreases the risk of overfitting. Overfitting is inevitable if the number of features exceeds the number of samples. Consider feature robustness when selecting features | - 3 (if neither measure is implemented)  +3 (if either measure is implemented) |
| **6** | **Multivariable analysis** with non radiomics features (for example, EGFR mutation) - is expected to provide a more holistic model. Permits correlating/inferencing between radiomics and non radiomics features | + 1 |
| **7** | Detect and discuss **biological correlates** - demonstration of phenotypic differences (possibly associated with underlying gene–protein expression patterns) deepens understanding of radiomics and biology | + 1 |
| **8** | **Cut-off analyses** - determine risk groups by either the median, a previously published cut-off or report a continuous risk variable. Reduces the risk of reporting overly optimistic results | + 1 |
| **9** | **Discrimination statistics** - report discrimination statistics (for example, C-statistic, ROC curve, AUC) and their statistical significance (for example, p-values, confidence intervals). One can also apply resampling method (for example, bootstrapping, cross-validation) | + 1 (if a discrimination statistic and its statistical significance are reported)  +1 (if a resampling method technique is also applied) |
| **10** | **Calibration statistics** - report calibration statistics (for example, Calibration-in-the-large/slope, calibration plots) and their statistical significance (for example, *P*-values, confidence intervals). One can also apply resampling method (for example, bootstrapping, cross-validation) | + 1 (if a calibration statistic and its statistical significance are reported)  +1 (if a resampling method technique is also applied) |
| **11** | **Prospective study** registered in a trial database - provides the highest level of evidence supporting the clinical validity and usefulness of the radiomics biomarker | + 7 (for prospective validation of a radiomics signature in an appropriate trial) |
| **12** | **Validation** - the validation is performed without retraining and without adaptation of the cut-off value, provides crucial information with regard to credible clinical performance | - 5 (if validation is missing)  +2 (if validation is based on a dataset  from the same institute)  +3 (if validation is based on a dataset from another institute)  +4 (if validation is based on two datasets from two distinct institutes)  +4 (if the study validates a previously published signature)  +5 (if validation is based on three or more datasets from distinct institutes)  *Datasets should be of comparable size and should have at least  10 events per model feature |
| **13** | **Comparison to ‘gold standard’** - assess the extent to which the model agrees with/is superior to the current ‘gold standard’ method (for example, TNM-staging for survival prediction). This comparison shows the added value of radiomics | +2 |
| **14** | **Potential clinical utility** - report on the current and potential application of the model in a clinical setting (for example, decision curve analysis). | +2 |
| **15** | **Cost-effectiveness analysis** - report on the cost-effectiveness of the clinical application (for example, QALYs generated) | +1 |
| **16** | **Open science and data** - make code and data publicly available. Open science facilitates knowledge transfer and reproducibility of the study | + 1 (if scans are open source)  + 1 (if region of interest  segmentations are open source)  + 1 (if code is open source)  + 1 (if radiomics features are calculated on a set of representative ROIs and the calculated features andrepresentative ROIs are open source) |
|  | Total points (36=100%) |  |

Source: https://www.radiomics.world/rqs

**Supplementary material S2:** Description of the revised Quality Assessment of Diagnostic Accuracy Studies (QUADAS-2) tool

| **Domain** | **Patient selection** | **Index test** | **Reference**  **standard** | **Flow and timing** |
| --- | --- | --- | --- | --- |
| **Signalling**  **questions**  **(yes, no, or**  **unclear)** | Was a consecutive or random sample of patients enrolled?  Was a case-control design avoided?  Did the study avoid inappropriate  exclusions? | Were the index test results interpreted  without knowledge of the results of the reference standard?  If a threshold was used, was it prespecified? | Is the the reference standard likely to correctly classify  the target condition?  Were the reference standard results interpreted without  knowledge of the results of the index test? | Was there an appropriate interval between index test  and reference standard?  Did all patients receive a reference standard?  Did all patients receive the same reference standard?  Were all patients included in the analysis? |
| **Risk of bias**  **(high, low,**  **or unclear)** | Could the selection of patients have  introduced bias? | Could the conduct or interpretation of the index test have introduced bias? | Could the reference standard, its conduct, or its interpretation have introduced bias? | Could the patient flow have introduced bias? |
| **Concerns**  **about**  **applicability**  **(high, low,**  **or unclear)** | Are there concerns that the included patients do not match the review question? | Are there concerns that the index test, its conduct, or its interpretation differ from the review question? | Are there concerns that the target condition as defined by the reference standard does not match the review question? | - |

Source: Whiting PF, Rutjes AW, Westwood ME, et al; QUADAS-2 Group. QUADAS-2: a revised tool for the quality assessment of diagnostic accuracy studies. Ann Intern Med. 2011 Oct 18;155(8):529-36. doi: 10.7326/0003-4819-155-8-201110180-00009.

|  | Risk of Bias |  |  |  | Applicability Concerns | | |
| --- | --- | --- | --- | --- | --- | --- | --- |
| Study ID | Patient  selection | Index test | Reference  standard | Flow and timing | Patient  selection | Index  test | Reference  standard |
| Avesani et al 2022 | Low | Low | Unclear | Low | Low | Low | Low |
| Du and Zhao 2022 | Low | Low | Low | Low | Low | Low | Low |
| Cao et al 2023 | Low | Low | Low | Low | Low | Low | Low |
| Deng et al 2024 | Low | Low | Low | Low | Low | Low | Low |
| Gierach et al 2014 | Low | Low | Low | Unclear | Low | Low | Low |
| Guo et al 2023 | Low | Low | Low | Low | Low | Low | Low |
| Huo et al 2002 | Low | Low | Low | Low | Low | Low | Low |
| Li et al (1) 2017 | Low | Low | Low | Unclear | Low | Low | Low |
| Li et al (2) 2021 | Low | Low | Low | Low | Low | Low | Low |
| Meier et al 2019 | Low | Low | Low | Low | Low | Low | Low |
| Mingzhu et al 2021 | Low | Low | Low | Low | Low | Low | Low |
| Nero et al 2020 | Low | Low | Low | Low | Low | Low | Low |
| Vasileiou et al 2020 | Low | Low | Low | Low | Low | Low | Low |

**Supplementary material S3:** Risk of bias and application concerns assessment of each study by the QUADAS-2 tool
